# Motor Learning in Response to Different Experimental Pain Models Among Healthy Individuals: A Systematic Review

**DOI:** 10.3389/fnhum.2022.863741

**Published:** 2022-03-24

**Authors:** Mohammad Izadi, Sae Franklin, Marianna Bellafiore, David W. Franklin

**Affiliations:** ^1^Sport and Exercise Research Unit, Department of Psychology, Educational Sciences and Human Movement, University of Palermo, Palermo, Italy; ^2^Institute for Cognitive Systems, Department of Electrical and Computer Engineering, Technical University of Munich, Munich, Germany; ^3^Neuromuscular Diagnostics, Department of Sport and Health Sciences, Technical University of Munich, Munich, Germany; ^4^Munich School of Robotics and Machine Intelligence, Technical University of Munich, Munich, Germany; ^5^Munich Data Science Institute, Technical University of Munich, Munich, Germany

**Keywords:** pain, exercise, rehabilitation, motor learning, adaptation

## Abstract

Learning new movement patterns is a normal part of daily life, but of critical importance in both sport and rehabilitation. A major question is how different sensory signals are integrated together to give rise to motor adaptation and learning. More specifically, there is growing evidence that pain can give rise to alterations in the learning process. Despite a number of studies investigating the role of pain on the learning process, there is still no systematic review to summarize and critically assess investigations regarding this topic in the literature. Here in this systematic review, we summarize and critically evaluate studies that examined the influence of experimental pain on motor learning. Seventeen studies that exclusively assessed the effect of experimental pain models on motor learning among healthy human individuals were included for this systematic review, carried out based on the preferred reporting items for systematic reviews and meta-analyses (PRISMA) statement. The results of the review revealed there is no consensus regarding the effect of pain on the skill learning acquisition and retention. However, several studies demonstrated that participants who experienced pain continued to express a changed motor strategy to perform a motor task even 1 week after training under the pain condition. The results highlight a need for further studies in this area of research, and specifically to investigate whether pain has different effects on motor learning depending on the type of motor task.

## Introduction

Pain is an unpleasant but important perception, in order to attract attention and avoid further damage to the body. However, patients and athletes are often required to learn new movement patterns as part of a rehabilitation program in the presence of pain conditions. While it may be necessary to perform rehabilitation exercise immediately after an injury in order to return to optimal performance, there is concern surrounding the effect of pain on the learning process. It has been reported that pain, as a sensory input, might affect the sensorimotor system leading to changes in motor performance, including redistribution of muscle activation patterns, and a reduction in muscle endurance that is essential for performing dynamic motor skills (Hodges and Tucker, 2011; Bank et al., 2013; Lamothe et al., 2014). In this vein, several studies (Dancey et al., 2014, 2016b; Mavromatis et al., 2017) demonstrated that pain can give rise to neuroplastic changes in the cortex. However, these neuroplastic changes have been associated with both decreases in motor performance (Mavromatis et al., 2017) and improvements in motor learning outcomes in response to pain (Dancey et al., 2016a).

In order to investigate the influence of pain on the learning process, experimental pain models, including muscle and cutaneous pain, have been used to test its effect on motor learning (Dancey et al., 2014, 2016a,^2019^; Lamothe et al., 2014). Other studies have examined the impact of chronic pain on motor learning (Vallence et al., 2013; Zaman et al., 2015). However, chronic pain cannot detect the pure influence of pain on this process, as chronic pain can also be associated with pain-related fear or tissue damage both of which could affect motor learning (Bank et al., 2013). Therefore, using only experimental pain models can assist in studying the pure effect of pain on the learning process.

To date, although many studies (Bouffard et al., 2014; Dancey et al., 2014, 2016a,b, 2019; Lamothe et al., 2014; Rittig-Rasmussen et al., 2014; Bilodeau et al., 2016) have examined the effect of experimental pain models on motor learning; they have provided contradictory findings. For example, some studies have suggested that acute cutaneous pain models improve motor learning acquisition (Dancey et al., 2014, 2016b) and retention (Dancey et al., 2016b), whereas Bilodeau et al. (2016) applied a similar experimental pain model and demonstrated no alteration in the learning process. Bouffard et al. (2016) also reported no alteration in motor performance in response to a similar experimental pain induction while those who experienced pain indicated distinct motor strategies compared to participants without pain performing a similar task.

Despite the growing literature on the knowledge of the learning process in the presence of experimental pain models, there has been no systematic study reviewing this literature. Considering the contradictory results related to motor learning during pain, it is important to synthesize and critically assess the studies on motor learning to assess experimental pain models. This information will help to a better understanding of the effect of pain on skill learning acquisition and retention, which is important for developing sport training and rehabilitation programs. Hence, the aim of the current study is to systematically review the research outputs that have examined the effect of experimental pain models (including muscle pain and cutaneous pain) on motor learning (including motor adaptation, motor performance, and motor strategy, but not neuroplasticity) among healthy human participants.

## Methods

This systematic review was reported based on the preferred reporting items for systematic reviews and meta-analyses (PRISMA) guidelines (Shamseer et al., 2015) and the protocol of the current review was registered in The International Prospective Register of Systematic Reviews (PROSPERO), registration number is CRD42020211489 (Izadi et al., 2020). A preprint of the present review is available on Medrxiv (Izadi et al., 2021).

### Search Strategy

Electronic databases (PubMed, Web of Science, and Embase) were used to search the literature up to April 2021. A combination of free-text terms and MeSH terms regarding motor learning (including retention) and experimental pain was applied (see Supplementary Material). Search strategies of relevant systematic reviews (Bank et al., 2013; Kim et al., 2017) were also checked in order to carry out an elaborate strategy. In addition, references of included studies were hand-searched to detect all pertinent studies, as well as the citations of the included studies were checked *via* Google Scholar.

### Eligibility Criteria

All studies that have the following criteria were included in this systematic review: (1) results of research from healthy human subjects; (2) experimental pain was induced in order to detect the effect of pain on the learning process; (3) original research with full text written in the English language; and (4) all study designs other than all types of reviews, meta-analysis, letter to editors, and theses. Studies that induced pain that can result in structural tissue damage, including pain with eccentric exercise and ischemia, were excluded from this study.

### Study Selection

Extraction of studies was performed by one reviewer, after which two authors independently reviewed retrieved titles and abstracts after removing duplicates. In the title and abstract screen phase, the two reviewers discussed any dispute regarding mismatch between their selections, and the full-text of any studies that were not agreed to be removed were considered for assessment in the second phase. Full-text was also reviewed by the two reviewers to ensure that studies were selected in accordance with the inclusion and exclusion criteria. In the case of disagreement between the two authors surrounding the inclusion or exclusion of a study in the full-text screen phase, the issue was resolved through consultations with a third reviewer. The two reviewers agreed on 3058 studies at the title and abstract screening stage, in which the full-text of 16 studies were directly agreed upon for full text screening. Discussion on a further 17 studies was done, out of which an additional 10 studies were selected to screen full-text. This resulted in 26 studies total for the full-text screening. Out of this number, 21 studies were agreed between the two reviewers at the full-text screening stage. The third reviewer consulted on the five other studies.

### Data Collection and Synthesis

In order to collect data, a standard form was used so that the following information was included in a table: (1) pain characteristics [location (i.e., the segment of pain induction), type, and intensity (i.e., mean pain)]; (2) outcome variables (i.e., parameters that were used to assess learning); (3) test protocol; (4) general information about characteristics of subjects; and (5) main results. One author gathered the mentioned data from all included studies and another author checked the collected data to decrease error and bias in data collection. A narrative synthesis was also applied to describe the collected data, which were categorized into cutaneous pain, muscle pain, and tongue pain. Furthermore, it was determined that it was not feasible to conduct a meta-analysis because of a methodological heterogeneity among the included studies. A qualitative synthesis, therefore, was considered for the current review.

### Quality Assessment

Two authors independently assessed the quality and bias of all included studies based on a modified version of the checklist for measuring the quality of RCTs (randomized controlled trials) and non-RCTs written by Downs and Black (1998) and Chuter et al. (2014). In the modified version of the checklist, item 27 (power) was changed from 0-5 to 0 or 1 so that a study was scored 1 if the study reported a statistical power ≥80%; otherwise, it received 0 (Chuter et al., 2014), so that the overall score of the checklist changed from 32 to 28. The quality of included studies was divided into the following four levels: excellent (26–28), good (20–25), fair (15–19), and poor (≤14) (Pas et al., 2015). Inter-rater reliability for the qualitative items was also measured by using the Kappa correlation coefficient between the two reviewers.

## Results

### Study Identification

**FIGURE 1:**
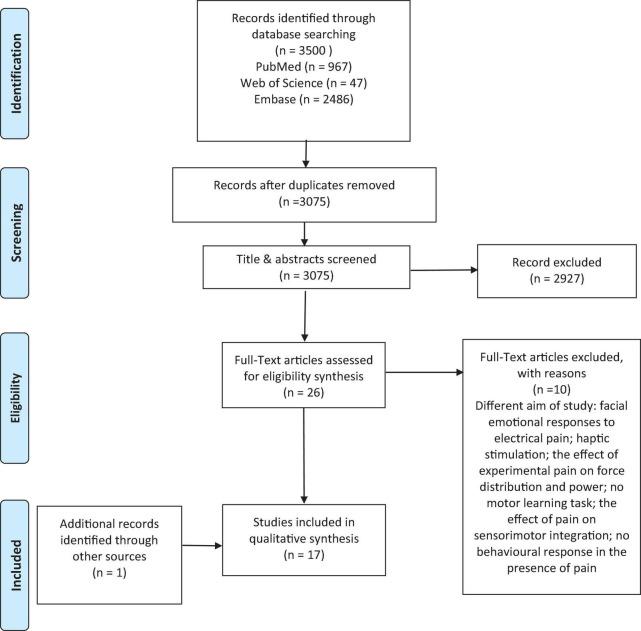
Flowchart of study selection process.

A total of 3500 articles were generated *via* electronic databases. The titles and abstracts of 3075 studies were screened after removing 425 duplicates. The full text of 26 studies (Bonifazi et al., 2004; Boudreau et al., 2007, 2010; Ingham et al., 2011; Bouffard et al., 2014, 2016, 2018; Dancey et al., 2014, 2016a,b, 2018, 2019; Lamothe et al., 2014; Rosati et al., 2014; Bilodeau et al., 2016; Boselie et al., 2016; Mancini et al., 2016; Mista et al., 2016; Brun et al., 2017; Mavromatis et al., 2017; Billot et al., 2018; Gallina et al., 2018, 2021; Alaiti et al., 2020; Schmidt et al., 2020; Arieh et al., 2021) were assessed in agreement with the inclusion and exclusion criteria in which only 16 studies (Boudreau et al., 2007, 2010; Ingham et al., 2011; Bouffard et al., 2014, 2016, 2018; Dancey et al., 2014, 2016a,b, 2018, 2019; Lamothe et al., 2014; Bilodeau et al., 2016; Mavromatis et al., 2017; Gallina et al., 2018; Arieh et al., 2021) were included in this review. Finally, one study (Salomoni et al., 2019) was added through a hand-searching of the citations of the relevant studies through Google Scholar; thus, 17 studies were included in this review (Figure 1).

### Study Characteristics

A total of 484 healthy participants were included across all studies with the ages ranging between 18 and 47 years. Out of the seventeen studies, Arieh et al. (2021) included only male participants, but both male and female participants were included in the other 16 studies (Boudreau et al., 2007, 2010; Ingham et al., 2011; Bouffard et al., 2014, 2016, 2018; Dancey et al., 2014, 2016a,b, 2018, 2019; Lamothe et al., 2014; Bilodeau et al., 2016; Mavromatis et al., 2017; Gallina et al., 2018; Salomoni et al., 2019). Extensive information concerning each study is represented in Table 1.

**TABLE 1:** Study characteristics.

References	Participants	Pain characteristics	Testing procedure	Outcome measures	Main results
Arieh et al. (2021)	Healthy (*N* = 30, *f* = 0) (remote pain, local pain, and control group, *N* = 10), age: 18–25 yrs	Capsaicin gel to the outer side of the elbow 5 cm (local pain); capsaicin gel to the upper part of the knee joint (remote pain); severity of pain was 7-measuring by VAS	Dart-throwing skill during acquisition (with pain) and retention (without pain) (1 h, 24 h, and 1 week) phases	Coordination variability pattern during throwing in both acquisition and retention phases (maximum wrist flexion range, maximum elbow extension range, shoulder angular displacement range, angular throw velocity, and throw duration)	No sig effect of pain on dart-throwing learning
Bilodeau et al. (2016)	Healthy (*N* = 45) (remote pain *N* = 15, *f* = 10, age: 28.5 ± 9.5 yrs); (local pain *N* = 15, *f* = 10, age: 27.4 ± 7.1 yrs); and (control group *N* = 15, *f* = 10, age: 28.8 ± 8.8 yrs)	Thermode 3 cm × 3 cm (heat pain) before acquisition phase to the dorsal part of the left wrist (local pain) and the external part of the left leg below the knee joint (remote pain); severity of pain was measured-measuring by NPRS	Finger-tapping task (reproducing the sequence 4-1-3-2-4) during 30 s	Error rate and speed of tapping sequences during baseline, post-immediate, post-60 min, and post-24 h (retention)	No sig effect of tonic pain on the acquisition and retention of finger-tapping task
Bouffard et al. (2014)	Healthy (*N* = 30) (pain group *N* = 15, *f* = 8, age: 26.0 ± 1.4 yrs); (control group *N* = 15, f = 7, age: 26.1 ± 2.1 yrs)	Capsaicin gel around the ankle prior to acquisition phase; severity of pain was moderate-measuring by NPRS	Walking task in the presence of a force field adaptation paradigm in 2 days [acquisition (baseline 1, baseline 2, adaptation, and wash-out) and retention (baseline, adaptation, and wash-out)]	A movement error signal that was made based on the ankle angular displacement	Sig effect of tonic pain on the retention phase of a locomotor task, while no sig change in the acquisition phase of gait
Bouffard et al. (2016)	Healthy (*N* = 37) (pain group *N* = 13, *f* = 8, age: 26.1 ± 1.15 yrs); (control group *N* = 24, *f* = 10, age: 25.8 ± 0.85 yrs)	Capsaicin gel around the ankle between baseline 1 and baseline 2 in the first day and prior to baseline in the second day; severity of pain was 5.6 ± 0.7 in Day 1 and 5.5 ± 0.7 in Day 2-measuring by NPRS	Walking task in the presence of a force field adaptation paradigm in 2 days [acquisition (baseline 1, baseline 2, adaptation, and wash-out) and retention (baseline, adaptation, and wash-out)]	A mean absolute error, which was created based on the ankle kinematics, and tibialis anterior ratios that showed TA muscle activation in the adaptation phase relative to baseline	No sig effect of cutaneous pain on total motor performance during both acquisition and retention phases
Bouffard et al. (2018)	Healthy (*N* = 47) (pain group *N* = 17, *f* = 7, age: 25 ± 1 yrs); (control group *N* = 30, *f* = 14, age: 25 ± 1 yrs)	Hypertonic saline to the tibialis anterior muscle prior to baseline 1 in the first day; the intensity of pain was 5.3 ± 1.2 out of 10-measuring by NPRS	Walking task in the presence of a force field adaptation paradigm in 2 days [acquisition (baseline 1, baseline 2, adaptation, and wash-out) and retention (baseline, adaptation, and wash-out)]	A mean absolute error, which was created based on the ankle kinematics; relative timing of ankle error; tibialis anterior ratios that showed TA muscle activation in the adaptation phase relative to baseline	No sig effect of muscle pain on total motor performance during both acquisition and retention phases
Dancey et al. (2016a)	Healthy (*N* = 24) (pain group *N* = 12, *f* = 8, age: 20.8 ± 3.3 yrs); (control group *N* = 12, *f* = 6, age: 22.8 ± 2 yrs)	Capsaicin gel for pain group and topical cream for the control group in the lateral part of the right elbow; pain intensity average was above 4-measuring by NPRS	Tracing sequences of sinusoidal pattern waves with various amplitudes and frequencies using the thumb in four phases [pre-acquisition, acquisition, post-acquisition, and retention (24–48 h later)]	Motor error which showed the average distance of subjects’ effort trace from the displayed sinusoidal wave	An improvement in motor learning in response to cutaneous pain
Dancey et al. (2016b)	Healthy (*N* = 48) (experiment 1 (*N* = 24; pain group *N* = 12, *f* = 7, age: 20.8 ± 3.3 yrs; control group *N* = 12, *f* = 6, age: 22.8 ± 2 yrs) (experiment 2 (*N* = 24; remote pain group *N* = 12, *f* = 7, age: 21.8 ± 3.3 yrs; local pain group *N* = 12, *f* = 7, age: 22.9 ± 4.3 yrs)	Capsaicin gel for pain group and topical cream for control group, remote pain and control pain in the lateral part of the dominant elbow and local pain in the Abductor Pollicis Brevis muscle area; pain intensity average was approximately six during post-motor learning-measuring by NRPS	A repetitive typing task	Response time and accuracy during a typing task at the begging and end of the motor acquisition and 48 h later (motor learning retention)	An improvement in motor learning retention in the presence of local pain; improved motor performance in the baseline in the presence of acute pain
Dancey et al. (2018)	Healthy (*N* = 36) (local pain group *N* = 12, *f* = 8, age: 21.2 ± 2.2 yrs); (remote pain group *N* = 12, *f* = 8, age: 20.3 ± 2.5 yrs); (contralateral pain group *N* = 12, *f* = 8, age: 21.4 ± 2.4 yrs)	Capsaicin gel for pain group in which remote and contralateral pain in the lateral part of the dominant and non-dominant elbow, respectively; local pain in the Abductor Pollicis Brevis muscle area; pain intensity average was evaluated-measuring by NPRS	Tracing sequences of sinusoidal pattern waves with various amplitudes and frequencies using thumb in four phases [pre-acquisition, acquisition, post-acquisition, and retention (24–48 h later)]	Motor error which showed the average distance of subjects’ effort trace from the displayed sinusoidal wave	No sig effect of pain location on motor learning acquisition and retention
Dancey et al. (2019)	Healthy (*N* = 24) (pain group *N* = 12, *f* = 9, age: 19.9 ± 0.9 yrs); (control group *N* = 12, *f* = 9, age: 20.7 ± 1.4 yrs)	Capsaicin gel for pain group and topical cream for control group in the lateral part of the dominant elbow; pain intensity average was above 4-measuring by NPRS	Tracing sequences of sinusoidal pattern waves with various amplitudes and frequencies using the thumb in four phases [pre-acquisition, acquisition, post-acquisition, and retention (24–48 h later)]	Mean motor error which showed the average distance of subjects’ effort trace from the displayed sinusoidal wave	An improvement in motor performance in the presence of tonic pain
Lamothe et al. (2014)	Healthy (*N* = 29) (pain group *N* = 15, *f* = 7, age: 25.8 ± 4.1 yrs); (control group *N* = 14, *f* = 8, age: 26.6 ± 4.8 yrs)	Capsaicin gel for pain group above the elbow between two baseline in the first day; pain intensity average was 7.8 ± 0.9 at the initiation of baseline 2-measuring by NPRS	A reaching task in the presence of force field adaptation in four phases [baseline 1, baseline 2, acquisition, and retention (24 h)]	Final error and the initial range of deviation	No sig effect of tonic pain on baseline reaching performance; a larger final error in the pain group than the control group during both acquisition and retention
Mavromatis et al. (2017)	Healthy (*N* = 30) (pain group *N* = 15, *f* = 6, age: 26 ± 6 yrs); (control group *N* = 15, *f* = 6, age: 27 ± 6 yrs)	Capsaicin gel for pain group around the lateral part of the first metacarpal after the first TMS baseline measurement; pain intensity average was above four at the training blocks-measuring by NPRS	A modified version of the sequential visual isometric pinch task in three phases (baseline 1, baseline 2, and acquisition)	Movement time, accuracy, and a skill measure	No sig effect of cutaneous pain on motor skill acquisition
Dancey et al. (2014)	Healthy (*N* = 24) (pain group *N* = 12, *f* = 7, age: 24.5 ± 6.6 yrs); (control group *N* = 12, *f* = 6, age: 23.4 ± 2 yrs)	Capsaicin gel for pain group and topical cream for control group in the lateral part of the right elbow; pain intensity average was 5 in the post-application phase-measuring by NPRS	A repetitive typing task applying the middle three fingers	Motor training accuracy; reaction time	An improvement in motor performance in the presence of tonic pain
Ingham et al. (2011)	Healthy (*N* = 9, *f* = 2, age: 24 ± 1.1)	Hypertonic saline for pain group; remote pain in the infrapatellar fat pad of the knee and local pain in the FDI; pain intensity average was 0.2 ± 0.4 (vehicle control), 1.7 ± 1 (FDI pain), and 2.1 ± 1.6 (remote pain)-measuring by NRS	A quick movements of finger (the right index finger) adduction	Training performance based on peak acceleration of index finger movement	No sig effect of pain on motor performance
Salomoni et al. (2019)	Healthy (*N* = 22) (pain group *N* = 11, *f* = 7, age: 24.5 ± 6.6 yrs); (control group *N* = 11, *f* = 5, age: 23.4 ± 2 yrs)	Hypertonic saline to the anterior deltoid muscle prior to baseline 2 and before the force field 1 in the first day; the intensity of pain was 4.2 ± 0.3 (out of 10) in the first injection and 3 ± 0.4 in the second injection-measuring by NPRS	A reaching task in the presence of force field adaptation in six phases (baseline 1, baseline 2, force field 1, force field 2, washout 1, force field 2, and washout 2)	Movement accuracy based on the peak perpendicular error and the peak hand velocity; muscle activity of anterior and posterior deltoid, biceps brachii, triceps brachii, and pectoralis major	No sig effect of pain on the final performance; experimental pain group used different strategies to perform the same task compared to the control group
Boudreau et al. (2007)	Healthy (*N* = 9, *f* = 2, age: 24 ± 1.1)	Capsaicin gel for pain group and vehicle cream for the control group to the tongue; pain intensity average was between 4 and 6 during the task-measuring by VAS	A tongue-protrusion task	A performance score based on the time that participants kept the cursor within the target box	A sig and negative effect of pain on the overall performance score
Boudreau et al. (2010)	Healthy (*N* = 26) (lidocaine group *N* = 9, *f* = 6, age: 24.6 ± 1.1 yrs); (control group *N* = 9, *f* = 3, age: 24 ± 3.5 yrs); (capsaicin group *N* = 8 *f* = 2, age: 23 ± 2.5 yrs)	Capsaicin and lidocaine gels for pain groups and vehicle cream for the control group to the dorsum of the tongue before the first task; pain intensity was maintained above 4-measuring by NRS	A tongue-protrusion task	(Overall, a tongue-task trial, initial, within-session gains) motor performance	A sig decrease in motor performance in the pain groups than the control group
Gallina et al. (2018)	Healthy (*N* = 14, *f* = 7, age: 18–47)	Hypertonic saline to the infrapatellar fat pad, distal vastus medialis, proximal vastus medialis, and vastus lateralis; the intensity of pain was approximately three in four different pain locations-measuring by NRS	Isometric knee extension contraction	Muscle activation of VMO, VL, and RF; isometric knee extension force	A sig alteration in both muscle activation and knee extension force in response to different locations of pain

*f, female; yrs, years; VMO, vastus medialis oblique; VL, vastus lateralis; RF, rectus femoris; sig, significant; VAS, visual analog scale; NRS, numerical rating scale; TMS, transcranial magnetic stimulation; FDI, first dorsal interosseus.*

### Methodological Quality

The methodological quality of the included studies was assessed based on the modified version of Downs and Black checklist, which is provided in Table 2. Out of 17 studies, 11 studies (Boudreau et al., 2010; Ingham et al., 2011; Bouffard et al., 2014, 2016; Dancey et al., 2014, 2016a,b, 2018, 2019; Bilodeau et al., 2016; Salomoni et al., 2019) were evaluated as fair quality and 6 articles as poor quality (Boudreau et al., 2007; Lamothe et al., 2014; Mavromatis et al., 2017; Bouffard et al., 2018; Gallina et al., 2018; Arieh et al., 2021). Inter-rater reliability was 0.72 between the assessors who evaluated the methodological quality of the included articles. Some of the items in the Downs and Black checklist may either be difficult for experimental studies on pain in motor learning to be assessed positively (e.g., item 13) or are often not reported in these experimental studies (e.g., items 11, 12, 19).

**TABLE 2:** Quality assessment of the included studies.

References	1	2	3	4	5	6	7	8	9	10	11	12	13	14	15	16	17	18	19	20	21	22	23	24	25	26	27	Total
Arieh et al. (2021)	1	1	1	1	1	1	1	0	0	1	U	U	0	U	U	1	1	1	U	1	U	1	1	U	0	U	0	14
Bilodeau et al. (2016)	1	1	1	1	2	1	1	0	0	1	U	U	0	U	U	1	1	1	U	1	U	1	1	U	0	U	0	15
Bouffard et al. (2014)	1	1	1	1	1	1	1	0	1	1	U	U	0	U	U	1	1	1	U	1	U	1	1	U	0	U	0	15
Bouffard et al. (2016)	1	1	1	1	1	1	1	0	1	1	U	U	0	U	U	1	1	1	U	1	1	1	1	U	0	U	0	16
Bouffard et al. (2018)	1	1	1	1	1	1	1	0	0	1	U	U	0	U	U	1	0	1	U	1	0	0	U	U	0	U	0	11
Dancey et al. (2016b)	1	1	1	1	1	1	1	0	0	1	U	U	0	1	U	1	1	1	U	1	1	1	U	U	0	U	0	15
Dancey et al. (2016a)	1	1	1	1	1	1	1	0	0	1	U	U	0	1	U	1	1	1	U	1	1	1	U	U	0	U	0	15
Dancey et al. (2018)	1	1	1	1	1	1	1	0	0	1	U	U	0	U	U	1	1	1	U	1	1	1	1	U	0	U	1	16
Dancey et al. (2019)	1	1	1	1	1	1	1	0	0	0	U	U	0	1	U	1	1	1	U	1	1	1	1	U	0	U	0	15
Lamothe et al. (2014)	1	1	1	1	1	1	1	0	1	1	U	U	0	U	U	1	1	1	U	1	U	1	U	U	0	U	0	14
Mavromatis et al. (2017)	1	1	1	1	1	1	1	0	0	1	U	U	0	U	U	1	1	1	U	1	U	1	1	U	0	U	0	14
Dancey et al. (2014)	1	1	1	1	1	1	1	0	0	1	U	U	0	1	U	1	1	1	U	1	1	1	1	U	0	U	0	16
Ingham et al. (2011)	1	1	1	1	1	1	1	0	1	1	U	U	0	U	U	1	1	1	U	1	U	1	1	U	0	U	0	15
Salomoni et al. (2019)	1	1	1	1	1	1	1	0	0	1	U	U	0	1	U	1	1	1	U	1	0	1	1	U	0	1	0	16
Boudreau et al. (2007)	1	1	1	1	0	1	1	0	0	1	U	U	0	1	U	1	U	1	U	1	U	U	1	U	0	U	0	12
Boudreau et al. (2010)	1	1	1	1	2	1	1	0	0	1	U	U	0	1	U	1	1	1	U	1	0	0	1	U	0	U	0	15
Gallina et al. (2018)	1	1	1	1	1	1	1	0	0	1	U	U	0	1	U	1	U	1	U	1	0	U	1	U	0	U	0	13

*U, unable to determine; 1, yes; 0, no. For item 5: 0, no; 1, partially; 2, yes.*

*Downs and Black checklist items: Reporting [(1) Is the hypothesis/aim/objective of the study clearly described?; (2) Are the main outcomes to be measured clearly described in the Introduction or Methods section?; (3) Are the characteristics of the patients included in the study clearly described?; (4) Are the interventions of interest clearly described?; (5) Are the distributions of principal confounders in each group of subjects to be compared clearly described?; (6) Are the main findings of the study clearly described?; (7) Does the study provide estimates of the random variability in the data for the main outcomes?; (8) Have all important adverse events that may be a consequence of the intervention been reported?; (9) Have the characteristics of patients lost to follow-up been described?; (10) Have actual probability values been reported (e.g., 0.035 rather than <0.05) for the main outcomes except where the probability value is less than 0.001?]; External validity [(11) Were the subjects asked to participate in the study representative of the entire population from which they were recruited?; (12) Were those subjects who were prepared to participate representative of the entire population from which they were recruited?; (13) Were the staff, places, and facilities where the patients were treated, representative of the treatment the majority of patients receive?]; Internal validity – bias [(14) Was an attempt made to blind study subjects to the intervention they have received?; (15) Was an attempt made to blind those measuring the main outcomes of the intervention?; (16) If any of the results of the study were based on “data dredging,” was this made clear?; (17) In trials and cohort studies, do the analyses adjust for different lengths of follow-up of patients, or in case-control studies, is the time period between the intervention and outcome the same for cases and controls?; (18) Were the statistical tests used to assess the main outcomes appropriate?; (19) Was compliance with the intervention/s reliable?; (20) Were the main outcome measures used accurate (valid and reliable)?]; Internal validity – confounding (selection bias) [(21) Were the patients in different intervention groups (trials and cohort studies) or were the cases and controls (case-control studies) recruited from the same population?; (22) Were study subjects in different intervention groups (trials and cohort studies) or were the cases and controls (case-control studies) recruited over the same period of time?; (23) Were study subjects randomized to intervention groups?; (24) Was the randomized intervention assignment concealed from both patients and health care staff until recruitment was complete and irrevocable?; (25) Was there adequate adjustment for confounding in the analyses from which the main findings were drawn?; (26) Were losses of patients to follow-up taken into account?]; (27) Power: Did the study have sufficient power to detect a clinically important effect where the probability value for a difference being due to chance is less than 5%?*

### Cutaneous Pain

Eleven studies (Bouffard et al., 2014, 2016; Dancey et al., 2014, 2016a,b, 2018, 2019; Lamothe et al., 2014; Bilodeau et al., 2016; Mavromatis et al., 2017; Arieh et al., 2021) applied capsaicin gel, resulting in cutaneous pain, to understand the effect of acute pain on motor learning. There was no consensus surrounding the effect of cutaneous pain on motor learning among these studies. Specifically, five studies (Bilodeau et al., 2016; Bouffard et al., 2016; Mavromatis et al., 2017; Dancey et al., 2018; Arieh et al., 2021) reported no significant change in motor performance in response to acute pain; however, some of them indicated alterations in the constructs of learning (Bouffard et al., 2016; Arieh et al., 2021). In contrast, six studies (Bouffard et al., 2014; Dancey et al., 2014, 2016a,b, 2019; Lamothe et al., 2014) demonstrated a statistically significant influence of cutaneous pain on motor learning. Each of the studies measured different motor learning variables applied during different motor tasks to study the effect of pain. In this context, Dancey et al. (2014, 2016a,b, 2018, 2019, 2014) carried out a series of studies to reveal the role of cutaneous pain on motor learning during typing and tracing series of sinusoidal patterns. The results of their studies show a statistically significant and positive influence of the experimental pain on skill acquisition learning (Dancey et al., 2014, 2016a,b, 2019) and retention (Dancey et al., 2016b,^2019^). While one study demonstrated that local pain improved retention of learning compared to remote pain (Dancey et al., 2016b), another study did not observe any significant effect on motor learning variables in response to different pain locations (remote, local, or contralateral) (Dancey et al., 2018). Two studies that used finger-tapping (Bilodeau et al., 2016) and sequential visual isometric pinch (Mavromatis et al., 2017) tasks to show the effect of cutaneous pain on motor learning found no significant differences in the pain group compared to the control group for both motor learning acquisition (Bilodeau et al., 2016; Mavromatis et al., 2017) and retention (Bilodeau et al., 2016). In addition, while Lamothe et al. (2014) indicated a significant improvement in motor performance in both control and pain groups during a new reaching adaptation task, the pain group demonstrated a larger final error to perform the same task compared to the control group in both acquisition and retention phases. Bouffard et al. (2014) revealed a significant decrease in the performance during the retention test of motor in the experimental cutaneous pain group during a novel locomotor adaptation task, with no difference between the groups during the initial learning. In a related study (Bouffard et al., 2016), they did not observe any considerable difference in either the learning or the retention of the new locomotor task, but in this study the capsaicin gel was applied in both the learning and retention tests. However, Bouffard et al. (2016) found that participants had a different pattern of kinematic errors in the presence of pain during walking (Table 3). Finally, Arieh et al. (2021) demonstrated no significant difference in movement accuracy in both acquisition and retention phases of motor learning in response to the experimental pain during dart-throwing skill. Nevertheless, participants in the pain group showed different coordination patterns in the shoulder–elbow and elbow–wrist joints to perform the task even 1 week later (Table 3).

**TABLE 3:** Synthesized results of the included studies.

References	Type of pain	Motor performance	Motor strategies
Arieh et al. (2021)	Cutaneous pain	No change in acquisition and retention phases	Change in coordination patterns
Bilodeau et al. (2016)	Cutaneous pain	No change in acquisition and retention phases	No report
Bouffard et al. (2014)	Cutaneous pain	No change in acquisition but a decrease in retention phase	No report
Bouffard et al. (2016)	Cutaneous pain	No change in acquisition and retention phases	Change in a pattern of kinematic errors
Bouffard et al. (2018)	Muscle pain	No change in acquisition and retention phases	Change in feedforward strategies
Dancey et al. (2016b)	Cutaneous pain	An increase in acquisition and no change in retention phases	No report
Dancey et al. (2016a)	Cutaneous pain	An increase in acquisition and retention phases	No report
Dancey et al. (2018)	Cutaneous pain	No change in acquisition and retention phases regarding pain location (local pain vs. remote pain)	No report
Dancey et al. (2019)	Cutaneous pain	An increase in acquisition and retention phases	No report
Lamothe et al. (2014)	Cutaneous pain	A decrease in acquisition and retention phases	larger final error to perform a reaching task
Mavromatis et al. (2017)	Cutaneous pain	No change in the acquisition phase	No report
Dancey et al. (2014)	Cutaneous pain	An increase in the acquisition phase	No report
Ingham et al. (2011)	Muscle pain	No change	No report
Salomoni et al. (2019)	Muscle pain	No change	Change even after the resolution of pain
Boudreau et al. (2007)	Tongue pain	A decrease in total performance	No report
Boudreau et al. (2010)	Tongue pain	A decrease in total performance	No report
Gallina et al. (2018)	Muscle pain	Change in total performance	Change in muscle activation pattern

### Tongue Pain

Boudreau et al. (2007, 2010) applied capsaicin gel to the tongue. The result of their studies revealed a significant negative influence of experimental cutaneous pain on overall motor performance during a tongue-protrusion task in a single day of training.

### Muscle Pain

Four studies (Ingham et al., 2011; Bouffard et al., 2018; Gallina et al., 2018; Salomoni et al., 2019) evaluated motor learning by injecting hypertonic saline, resulting in muscle pain, during different tasks. All studies revealed no significant effect of experimental muscle pain on motor performance. Specifically, Bouffard et al. (2018) did not observe any statistically significant change in acquisition and retention of a novel locomotor adaptation task in response to the experimental pain. However, motor strategies were different in those who experienced pain compared to the control group such that subjects with pain less depended on feedforward strategies than subjects without pain (Table 3). Ingham et al. (2011) also demonstrated no significant effect on motor learning in a finger adduction task in which muscle pain was applied in different locations. While Salomoni et al. (2019) did not observe any statistically significant alteration in final motor performance in the pain group compared to the control group during a reaching adaptation task, those who experienced the experimental muscle pain applied a distinct strategy to perform the task in comparison with the control group. The experimental group also produced the same strategy in the next day in the absence of pain. A similar result was found by Gallina et al. (2018) in which the muscle pain location produced lasting changes in the muscle activation pattern during an isometric knee extension contraction task.

## Discussion

The aim of the current study was to understand the effect of acute pain on motor learning among healthy individuals. Inconsistent results have been reported surrounding this topic in the literature; however, most of these studies are in agreement with the negative consequences of acute pain in the learning process. Moreover, while some studies did not demonstrate any significant effect of experimental pain on skill learning acquisition and retention, they indicated those who experienced pain produced a distinct strategy to perform the novel task compared to control groups such that the participants displayed the same strategy in pain resolution even after 1 week.

Learning new movement patterns is an integral part of sport and rehabilitation (Franklin and Wolpert, 2011), while pain can give rise to alterations in the learning process. Results of the studies that have examined the effect of experimental pain on motor learning corroborate the role of acute pain on changes in the learning process; however, the studies demonstrated contradictory findings. Specifically, a series of investigations by Dancey et al. (2014, 2016a,b, 2019) revealed a positive and statistically significant effect of cutaneous pain on the learning process. It has been suggested that pain can lead to an increase in attention while performing a dynamic task thereby those who experience pain can execute a function with lesser errors than no pain condition (Hazeltine et al., 1997; Dancey et al., 2016b). In this context, Dancey et al. (2014, 2016a,b, 2018) reported an improvement in skill learning acquisition and retention in the presence of experimental pain because of attention mechanism, in which local pain brought about a better overall motor performance compared to remote pain. It was argued that local pain may result in more attention to the part of the body (i.e., internal attention) underlying learning (Dancey et al., 2016b), which in turn can lead to more changes in cortical neuroplasticity (Rosenkranz and Rothwell, 2004), and subsequently improve in the learning of motor tasks. However, a recent study (Tumialis et al., 2020) indicated that external attention can engender more accuracy and better performance in comparison to internal attention, including performing a task in the presence of pain. Whereas Mavromatis et al. (2017) applied a similar experimental pain model compared to the work of Dancey et al. (2014, 2016a,b, 2018), they did not observe any significant effect of acute pain on the skill learning acquisition. However, the training-related alterations in corticospinal excitability were showed a similar result to Dancey’s study in the presence of cutaneous pain (Mavromatis et al., 2017). In contrast to the previous studies, Boudreau et al. (2007, 2010) reported a significant negative influence of cutaneous pain on overall performance scores. While all of these studies applied a similar experimental tonic pain, the studies used a range of tasks to understand the effect of pain on motor learning which might explain some of the differences in the findings. In particular, different motor tasks and different types of learning depend on different brain mechanisms and brain areas (Doya, 2000; Ghilardi et al., 2000). These differences may actually be one of the major reasons why we find inconsistent results of the effect of pain on motor learning (Bilodeau et al., 2016). For example, force field adaptation and sequence learning tasks can rely on cortico-cerebellar and cortico-striatal plasticity, respectively (Doyon and Benali, 2005). Similarly, Seidler et al. (2004) found large differences in brain activation even within a similar task; where performance with smaller errors during movements to large easy to reach targets were associated with greater activation in the contralateral primary motor cortex, premotor cortex and the basal ganglia, and larger errors during movements to small targets associated with greater activation in the ipsilateral motor cortex, insular cortex, cingulate motor area, and multiple cerebellar regions. That is, variations in the task difficulty (e.g., target size) can influence the degree of feedforward relative to feedback control that contributes to the task performance, and therefore the specific brain areas involved. It is very likely that the different circuitry and adaptation mechanisms involved in different motor tasks have different reactions to painful stimuli.

While the studies that applied cutaneous pain to evaluate motor learning revealed contradictory results, most research examining experimental muscle pain on motor learning outcomes found no significant effect on motor performance while revealing alterations in motor strategies (Ingham et al., 2011; Bouffard et al., 2018; Salomoni et al., 2019). Indeed, it has been reported that these two experimental pain models interact distinctly with neural processes that are responsible for motor adaptation (Henderson et al., 2006), which in turn can lead to observe different results in skill learning acquisition and retention in response to cutaneous or muscle pain models. This distinction between cutaneous and muscle pain models was particularly clear in a series of studies by Bouffard et al. (2014); Burdet et al. (2013); Bouffard et al. (2018) demonstrating motor learning outcomes in response to experimental muscle and cutaneous pain models during a locomotor adaptation task. Specifically, there was no alteration in skill learning acquisition or retention in the presence of experimental muscle pain (Bouffard et al., 2018). However, they did find a statistically significant reduction in retention (but not acquisition) of the same test in the presence of the cutaneous pain model (Bouffard et al., 2014). However, a follow-up study showed that cutaneous pain had no effect on either the acquisition or the retention as long as this pain was also applied during the test for retention (Bouffard et al., 2016). That is, it appeared that the cutaneous pain acted as a contextual signal for the selection of the newly learned locomotion model (Bouffard et al., 2016), similar to the manner that visual, proprioceptive and vestibular signals can be used to learn and recall different motor memories (Howard et al., 2012; Sarwary et al., 2015; Howard and Franklin, 2016). Although no considerable influence of cutaneous pain on motor performance or motor learning was shown, Bouffard and colleagues reported that participants in the pain group produced a distinct strategy compared to the control group to perform a locomotion task. Specifically, they found that participants had a different pattern of kinematic errors in the presence of pain during walking suggesting the pain group used less predictive compensation (anticipatory strategies) for the changes in the task (Bouffard et al., 2016). This finding was supported by several other studies (Bouffard et al., 2018; Salomoni et al., 2019; Arieh et al., 2021) which found participants exposed to experimental pain expressed a different strategy for motor adaptation compared to control participants despite no significant change in overall motor performance in the skill learning acquisition and retention. Notably, Salomoni et al. (2019) found that participants who experienced experimental muscle pain produced less co-contraction and muscle activation of the elbow and shoulder muscles compared to the pain-free control group during a reaching task, and this distinct motor strategy was continued on the next day (retention) despite the pain no longer being present. This smaller muscle co-contraction could potentially reduce joint stability in the coordination of musculoskeletal system (Franklin et al., 2007, 2013) and subsequently increase the potential for musculoskeletal injuries during sport training and rehabilitation (Henriksen et al., 2007). Arieh et al. (2021) also showed a similar motor performance in response to experimental pain compared to the control group during dart-throwing skill; however, participants in the pain group showed different coordination patterns in the shoulder–elbow and elbow–wrist joints to perform the task even 1 week later. These different movement patterns may be a strategy to decrease pain while still performing the motor adaptation task, as suggested by Hodges and Tucker (2011), in which pain affects the redistribution of activity within and between muscles (Muceli et al., 2014; van den Hoorn et al., 2015) to perform a motor task with a pain-free movement pattern. While this mechanism might be used to reduce pain during the learning process, such an alteration could potentially be associated with repercussions for the health condition of joints over longer time periods. That is, redistribution of muscle function can bring about changes in natural biomechanics of the joints by increasing joint load (Hodges and Tucker, 2011). These changed patterns of muscle activation or joint coordination can then persist over long periods of time either due to use-dependency (Doya, 2000; Diedrichsen et al., 2010; Burdet et al., 2013) or because the adaptation process resulted in local minimum of the solution space (Burdet et al., 2013).

### Limitations and Recommendations

The results of the present systematic review need to be interpreted with the consideration of the following methodological issues. In terms of experimental pain models, the International Association for the study of pain has suggested considering sex and gender differences in pain investigations (Greenspan et al., 2007) since women exhibit higher pain sensitivity in response to numerous pain conditions compared to men (for review, see Alabas et al., 2012; Bartley and Fillingim, 2013). Despite this, none of the included studies (Boudreau et al., 2007, 2010; Ingham et al., 2011; Bouffard et al., 2014, 2016, 2018; Dancey et al., 2014, 2016a,b, 2018, 2019; Lamothe et al., 2014; Bilodeau et al., 2016; Mavromatis et al., 2017; Gallina et al., 2018; Salomoni et al., 2019; Arieh et al., 2021) reported sex differences in motor learning outcomes in response to experimental pain models. Hence, it can be difficult to exclusively generalize the findings of the current systematic review to each sex and gender. As a result, it is recommended that further studies specify the influence of experimental pain modalities on motor adaptation and performance in regard to sex and gender differences. Aside from the former issue, it has been proposed that the experimental pain sensitivity can be altered across the menstrual cycle (Riley et al., 1999); therefore, future investigations need to consider this element as a confounder, which in turn can lead to affect the result of studies and to hardly interpret the alterations of motor learning variables in response to experimental pain models.

Studies that were included in the current review applied different experimental pain models. Two investigations (Henderson et al., 2006; Burton et al., 2009) demonstrated that cutaneous pain evokes distinct emotional and perceptual responses compared to deep pain. In particular, it has been reported that superficial pain can only be perceived surrounding the site of injection, whereas deep pain can diffuse to the other adjacent segments. Moreover, different cardiovascular and behavioral responses were observed in regard to the origin of pain (superficial or deep) (Henderson et al., 2006; Burton et al., 2009). These differences may lead to affect the result of pain studies. For example, several studies (Ingham et al., 2011; Bouffard et al., 2018; Gallina et al., 2018; Salomoni et al., 2019) that applied hypertonic saline injection to induce muscle pain did not report the depth of the injections, which potentially makes it difficult to generalize the effects of experimental muscle pain on motor learning variables. A very superficial injection might be more similar to cutaneous pain, whereas deeper injections may produce pain over wider areas. Thus, it is suggested that further studies report the depth of the hypertonic saline injection. Moreover, the aforementioned experimental pain models are continuously affected by any movement or posture of the subjects. That is, while hypertonic saline injection and capsaicin pain models – as tonic pain – can help to understand neurophysiological processes responsible for pain adaptation, perceived pain can be exacerbated or alleviated by specific movements or postures among those who experience musculoskeletal pain. This could lead to an alteration of motor adaptation when pain is increased or decreased (Lewis, 2016; Madry et al., 2016; Gallina et al., 2021). In this vein, a recent study by Gallina and colleagues proposed a new experimental pain model whereby pain can be modulated in regard to changes in movement and posture. Specifically, a low-frequency sinusoidal electrical stimulation has been suggested as a task-relevant pain in order to unravel the previously mentioned limitation (Gallina et al., 2021).

While the included studies applied visual analogue scale (VAS) or numeric rating system (NRS) to measure pain intensity, each study varied the evaluation methods, which can affect the interpretations of research findings (Smith et al., 2015). In addition, none of the included studies assessed the pain intensity at specific times throughout the experiment, or reported information such as assessment frequency, endpoint, or anchors that are important to reproduce such studies (for review, see Smith et al., 2015). Moreover, stress arising from injection could lead to increase pain sensitivity in particular subjects (Cathcart et al., 2008), such that studies in which non-painful injections were applied for control group could still result in stress and affect baseline pain sensitivity. This could make it difficult to interpret the pure influence of experimental pain models on motor learning variables, without controlling this potential confound. This is, the studies that applied non-painful injection, including isotonic saline, in the control group, did not report stress measurements in this group, and future investigations need to also consider the stress from injection and to precisely manage pain intensity evaluation across all conditions. Pain intensity is also of great interest for further studies in which to understand the effect of decreased or increased pain intensity on motor adaptation. More specifically, any simple correlation between anticipated sensory input and behavioral output is challenged by taking into consideration the nature of relief (Seymour et al., 2005). For instance, mild pain will be rewarding if it immediately comes after severe pain. In this manner, Seymour et al. (2005) demonstrated the possible neural process for pain relief in the upper motor level in which pain and relief related-expectancies were reported that can result in a strong impact on the following experience of actual pain. Moreover, there have been reported that several psychological factors, including depression (Dickens et al., 2003; Thompson et al., 2016), social support (for review, see Che et al., 2018), and sleep deprivation (Stroemel-Scheder et al., 2020) can affect pain perception and intensity. None of the included studies reported these elements, which could have influenced the results of the research.

Since there are reports that ethnicity, race, and culture may bring about different pain perceptions (for review, see Kim et al., 2017; Meeus, 2018), it is impossible to generalize the results of the current systematic review to different racial and ethnic groups. The individual differences in pain perception (Paine et al., 2009; Kobuch et al., 2016) also pose a question whether personal differences can trigger different responses in motor learning variables concerning experimental pain models. Nonetheless, none of the included studies demonstrated inter-subject responses to motor skill acquisition and retention in the presence of experimental pain models. Future studies are needed to examine whether there are significant differences between individuals regarding motor learning variables in response to experimental pain models. Wide individual variability could limit our ability to detect a major group effect of pain on motor adaptation.

In terms of motor learning, sleep between the acquisition and retention phases can be a factor that also influences motor learning, and only one study (Bilodeau et al., 2016) considered this issue before evaluating motor learning in response to experimental pain. Moreover, as physical and mental performances can fluctuate due to circadian rhythm; it has been suggested that physical and mental tests should be measured at the same time of day, especially for studies that apply repeated measurement protocols (Vitale and Weydahl, 2017). None of the studies reported this possible factor when assessing motor learning in response to experimental pain models. Furthermore the difficulty of a new motor adaptation task can result in a challenge to the success of performing a task (Guadagnoli and Lee, 2004); hence, the optimal challenge point should be determined when designing a motor learning task, to ensure that sufficient outcome measurement sensitivity is obtained. Otherwise, if the tasks are too difficult, too simple, or the performance measurement is too imprecise, a study may find no difference between control and pain groups even when a difference actually exists, producing a type 2 error (false negative). None of the included studies mentioned this important issue. In addition, only one study (Dancey et al., 2018) reported an adequate sample size for carrying out its research. Finally, the studies included in this review particularly focused on the effect of pain on motor learning in young healthy individuals, so further studies are needed to verify if similar effects are found in children and older adults, as well as expanding to patients and chronic pain conditions.

The current systematic review also has several limitations. First, only studies that were published in English language were considered to be reviewed while researches with other languages were not included in the present systematic review. Second, we did not include any theses or dissertations in our review. While including dissertations can help to decrease the potential publication bias (a bias that may arise from the fact that those results that are statistically significant are more likely to be published), theses have not been peer reviewed. Here we focused only on including peer reviewed and published literature. Third, pain can trigger alterations in the construct of learning, which in turn can lead to neuroplastic changes in the cortex; however, we only reported data regarding behavioral response in the presence of experimental pain models, and excluded studies that focused only on neural plasticity. While a recent systematic (Rohel et al., 2021) examined the effects of pain on corticospinal excitability, there is still an open question regarding the general effects of pain on neural plasticity induced during motor learning. Finally, the heterogenous nature of the included studies did not allow us to perform a meta-analysis, and therefore a narrative synthesis of the included studies was done instead.

## Conclusion

Overall, this systematic review found heterogeneous results regarding experimental pain models’ influence on motor learning. In particular, although experimental pain models have been reported to lead to changes in the skill learning acquisition and retention, many studies have also shown unaltered adaptation in motor learning outcomes. Finally, several studies have shown that distinct strategies have been observed in the pain group even after pain resolution. These variable results highlight the need for further studies to clarify the effect of pain on motor learning.

## Data Availability Statement

The original contributions presented in the study are included in the article/Supplementary Material, further inquiries can be directed to the corresponding author.

## Author Contributions

MI and DF contributed to study design and writing the first draft. SF, MI, and MB contributed to data collection, synthesizing the data, and searching the literature. All authors contributed to revising the manuscript and preparing the final version of the manuscript.

## Conflict of Interest

The authors declare that the research was conducted in the absence of any commercial or financial relationships that could be construed as a potential conflict of interest.

## Publisher’s Note

All claims expressed in this article are solely those of the authors and do not necessarily represent those of their affiliated organizations, or those of the publisher, the editors and the reviewers. Any product that may be evaluated in this article, or claim that may be made by its manufacturer, is not guaranteed or endorsed by the publisher.
