# Supplementary material for: Motor Learning in Response to Different Experimental Pain Models Among Healthy Individuals: A Systematic Review
Source: Front Hum Neurosci. 2022 Mar 24;16:863741. doi: 10.3389/fnhum.2022.863741 (PMC8987932; doi:10.3389/fnhum.2022.863741)
Supplement: Supplementary file 1 [file Data_Sheet_1.docx]

***Supplementary material***

| **#1** | (“motor learning”[tw] OR “motor adaptation”[tw] OR (skill [tw] OR acquisition [tw] OR learning [tw] OR retention[tw]) OR “motor behavior” [tw] OR “motor training” [tw] OR “muscle memory” [tw] OR "Learning"[Mesh] OR "Motor Skills"[Mesh] OR "Retention, Psychology/physiology"[Mesh] ) |
| --- | --- |
| **#2** | (“experimental muscle pain”[tw] OR “experimental pain”[tw] OR “pain stimul*”[tw] OR “pain threshold”[tw] OR “pain tolerance”[tw] OR "cutaneous stimul*"[tw] OR “cutaneous pain”[tw] OR “mechanical cutaneous pain”[tw] OR “thermal stimul*”[tw] OR “thermal pain”[tw] OR “heat pain”[tw] OR “heat stimul*”[tw] OR “heat tolerance”[tw] OR “thermal threshold”[tw] OR “pressure pain“[tw] OR “mechanical pain”[tw] OR “mechanical pressure”[tw] OR “mechanical stimul*”[tw] OR “chemical pain”[tw] OR “chemical stimul*”[tw] OR “cold pain”[tw] OR “cold stimul*”[tw] OR “cold tolerance”[tw] OR “nociceptive stimul*”[tw] OR “electrical pain”[tw] OR ((“hypertonic saline”[tw] OR “Saline Solution, Hypertonic”[mesh] OR “capsaicin”[tw] OR "Capsaicin"[Mesh] OR ”intramuscular glutamate”[tw] OR “laser evoked potential*”[tw]) AND pain) OR (“induced pain”[tw] OR “pain induced”[tw])) |
|  | ((#1 AND # 2) NOT ("Animals"[Mesh] NOT "Humans"[Mesh]) |

Table 1. Search strategy for PubMed

| **#1** | TS=(“motor learning” OR “motor adaptation” OR (skill OR acquisition OR learning OR retention) OR “motor behavior” OR “motor training” OR “muscle memory”) |
| --- | --- |
| **#2** | TS=(“experimental muscle pain” OR “experimental pain” OR “pain stimul*” OR “pain threshold” OR “pain tolerance” OR "cutaneous stimul*" OR “cutaneous pain” OR “mechanical cutaneous pain” OR “thermal stimul*” OR “thermal pain” OR “heat pain” OR “heat stimul*” OR “heat tolerance” OR “thermal threshold” OR “pressure pain“ OR “mechanical pain” OR “mechanical pressure” OR “mechanical stimul*” OR “chemical pain” OR “chemical stimul*” OR “cold pain” OR “cold stimul*” OR “cold tolerance” OR “nociceptive stimul*” OR “electrical pain” OR ((“hypertonic saline” OR “capsaicin” OR ”intramuscular glutamate” OR “laser evoked potential*”) AND pain) OR (“induced pain” OR “pain induced”)) AND TS=(human* OR homonid*) |
|  | #1 AND # 2 |

Table 2. Search strategy for Web of Science

| **#1** | (“motor learning”.mp OR “motor adaptation”.mp OR (skill .mp OR acquisition .mp OR learning .mp OR retention.mp) OR “motor behavior” .mp OR “motor training” .mp OR “muscle memory” .mp OR "learning"/ OR "motor skills"/ OR "retention, psychology/physiology"/) |
| --- | --- |
| **#2** | (“experimental muscle pain”.mp OR “experimental pain”.mp OR “pain stimul*”.mp OR “pain threshold”.mp OR “pain tolerance”.mp OR "cutaneous stimul*".mp OR “cutaneous pain”.mp OR “mechanical cutaneous pain”.mp OR “thermal stimul*”.mp OR “thermal pain”.mp OR “heat pain”.mp OR “heat stimul*”.mp OR “heat tolerance”.mp OR “thermal threshold”.mp OR “pressure pain“.mp OR “mechanical pain”.mp OR “mechanical pressure”.mp OR “mechanical stimul*”.mp OR “chemical pain”.mp OR “chemical stimul*”.mp OR “cold pain”.mp OR “cold stimul*”.mp OR “cold tolerance”.mp OR “nociceptive stimul*”.mp OR “electrical pain”.mp OR ((“hypertonic saline”.mp OR sodium chloride/ OR “capsaicin”.mp OR "capsaicin"/ OR ”intramuscular glutamate”.mp OR “laser evoked potential*”.mp) AND (exp pain OR pain.mp)) OR (“induced pain”.mp OR “pain induced”.mp)) |
|  | ((#1 AND # 2) NOT (animal NOT human) |

Table 3. Search strategy for EMBASE
